# Brain tumor image generation using an aggregation of GAN models with style transfer

**DOI:** 10.1038/s41598-022-12646-y

**Published:** 2022-06-01

**Authors:** Debadyuti Mukherkjee, Pritam Saha, Dmitry Kaplun, Aleksandr Sinitca, Ram Sarkar

**Affiliations:** 1 Department of Computer Science and Engineering, Jadavpur University, Kolkata, 700032 India; 2 Department of Electrical Engineering, Jadavpur University, Kolkata, 700032 India; 3 Department of Automation and Control Processes, Saint Petersburg Electrotechnical University “LETI”, Saint Petersburg, 197022 Russian Federation

**Keywords:** Image processing, Magnetic resonance imaging, Cancer imaging

## Abstract

In the recent past, deep learning-based models have achieved tremendous success in computer vision-related tasks with the help of large-scale annotated datasets. An interesting application of deep learning is synthetic data generation, especially in the domain of medical image analysis. The need for such a task arises due to the scarcity of original data. Class imbalance is another reason for applying data augmentation techniques. Generative Adversarial Networks (GANs) are beneficial for synthetic image generation in various fields. However, stand-alone GANs may only fetch the localized features in the latent representation of an image, whereas combining different GANs might understand the distributed features. To this end, we have proposed AGGrGAN, an aggregation of three base GAN models—two variants of Deep Convolutional Generative Adversarial Network (DCGAN) and a Wasserstein GAN (WGAN) to generate synthetic MRI scans of brain tumors. Further, we have applied the style transfer technique to enhance the image resemblance. Our proposed model efficiently overcomes the limitation of data unavailability and can understand the information variance in multiple representations of the raw images. We have conducted all the experiments on the two publicly available datasets - the brain tumor dataset and the Multimodal Brain Tumor Segmentation Challenge (BraTS) 2020 dataset. Results show that the proposed model can generate fine-quality images with maximum Structural Similarity Index Measure (SSIM) scores of 0.57 and 0.83 on the said two datasets.

## Introduction

The age of Artificial Intelligence (AI) has brought many opportunities to make our living standards better. Medical diagnosis and healthcare facilities are benefited from the perks of AI as well. Various deep learning techniques are proven to be helpful for medical image analysis which further lead to a better understanding of diseases occurring in the human body^1^. Brain tumor scan comes under the category of medical imaging domain. tumors are formed due to the excessive growth of cells that occurred in a particular region of the human body including the brain region. Proper identification of early-stage brain tumor is necessary because tumor formation in the brain may ultimately cause long-term disability in our body^2^ whereas, severe cases of brain tumor such as High-Grade Glioma (HGG) may ultimately result death^3^. A huge number of brain tumor cases has been reported in the USA in the last few years, and many succumbed to death^4^. Considering the severity caused by the brain tumor, many researchers developed computer-based diagnosis systems using deep learning models for screening brain tumor scan images. The most popular way of obtaining these brain scans is Magnetic Resonance Imaging (MRI) in which a magnetic field is applied to detect the presence of the tumor in the brain. MRI is also able to estimate the size of the present tumor. Although MRI has its limitations like consuming too much time or causing Claustrophobia, it is preferred over other methods for brain tumor detection due to its overall performance^5^. Being a powerful non-invasive analytic tool, MRI is applied to the image-based diagnosis of various systems throughout the body^6,7^.

MRI of a brain tumor can provide some key information, such as location, size, shape, irregularity, the intra-tumoral structure of brain tumors to physician by qualitative or quantitative analysis^8,9^, which help to identify the growth state of brain tumors and evaluate the performance of analysis. In this regard it is to be noted that for better training a machine learning or deep learning model, sufficient volume of data is necessary^10^, which is also true for this kind of medical image analysis. Data with high-class imbalance or insufficient variability leads to poor analytical results^11,12^. There are some publicly available datasets for conducting research in various medical image analysis, however in many cases, they are either small in size or due to privacy concerns, common researchers cannot have the access to the datasets. Data augmentation is a commonly used approach in this field to overcome the problem of limited datasets. Basic image processing operations can be used for generating new images. However, such techniques may not be very effective in the medical imaging domain since it lacks the ability to add the sufficient information to generated images in order to make it natural. Simple image augmentation techniques are also unable to understand the underlying features of the image. Besides, image manipulation techniques such as translation and rotation might change the pattern useful for the diagnosis. These datasets contain highly correlated image training data^12^. Hence, machine learning models trained on these augmented data gain little performance improvements due to the lack of variance of the data. Another type of data augmentation strategy adopted in literature is synthetic data generation. A synthetic dataset can be generated using computer-based programs. Such datasets can be highly beneficial for the purpose of medical image analysis. Furthermore, in this case there is no patient data handling or privacy concerns as the samples are produced synthetically^1^.

GANs^13^ can be used to generate synthetic data with good generalization ability and also serve as an effective method of data anonymization. GAN has two different networks—Generator and Discriminator. The model is trained in an adversarial process, where the Generator generates fake images, and the Discriminator learns to discriminate between the real and fake images^1^. GAN can generate synthetic images which can be used to share the data outside of the institution, to do different medical analysis and also can be used as an anonymization tool^12^.

Generation of medical images especially brain tumor scans is a challenging task overall. Traditional image augmentation techniques such as translation, rotation, scale, flip, etc. cannot produce sufficient variation in shape, location, size of a tumor in brain scans^14^. However, in the recent past, GANs have been used to perform the image generation task in various domains quite successfully. GAN models have yielded better outcomes for both generation and segmentation tasks on brain tumor scan images in several works performed previously. Shin et al.^11^ have segmented the overall scans of Alzheimer’s Disease Neuroimaging Initiative (ADNI)^15^ dataset and BraTS dataset^16^ into brain anatomy, tumors using pix2pix^17^ GAN. They have obtained the augmented scans by applying different combinations of the segmented brain anatomy and tumor labels by introducing some alternations. Islam et al.^1^ have proposed a Deep Convolutional Generative Adversarial Network (DCGAN)^18^ model for generating synthetic Positron emission tomography (PET) scan images from the scans present in the ADNI^15^ dataset. They have synthesized scans for all the three different stages of Alzheimer’s disease—Normal Control (NC), Mild Cognitive Impairment (MCI) and Alzheimer’s Disease (AD).

Han et al.^19^ have applied DCGAN and WGAN separately^20,21^ on the BraTS 2016 dataset^16^ to generate artificial MRI scans. To validate their results, they have conducted the Visual Turing Test on 50 real, 50 fake images and have achieved the highest performance for the WGAN with 53% accuracy. Lei et al.^22^ have proposed Dense cycle GAN to generate Computed Tomography (CT) scans from MRI scans based on one-to-one mapping formed during the training procedure. They have collected the original MRI and CT scans from 24 brain cancer patients and 20 prostate cancer patients. Finally, they have achieved the following results: (i) Mean Absolute Error (MAE) as 55.7 Hounsfield Units (HU) and 50.8 HU, (ii) mean Peak Signal-to-Noise Ratio (PSNR) as 26.6 dB and 24.5 dB, (iii) Normalized Cross-Correlation (NCC) as 0.963 and 0.929 in the brain cancer and prostate cancer scans respectively. GAN has shown outstanding performance in several research works conducted on image segmentation tasks as well such as Li et al.^14^ have proposed TumorGAN on the BraTS 2017 dataset^16^.

Nie et al.^23^ have used Fully Convolutional Network (FCN)^24^ as the generator and a basic CNN as the discriminator. They have proposed a basic 3D FCN to estimate the target image from the corresponding source image. They have used ADNI^15^ dataset and pelvic dataset (an in-house dataset) as the source images. They have obtained a mean PSNR of 34.1 and MAE of 39.1 for their proposed method. Emami et al.^25^ have proposed a GAN-based model where ResNet^26^ is used as the generator and discriminator is a CNN with five convolutional layers that have classified the input image as real or fake. They have achieved a mean PSNR of 26.6 ± 1.2 and SSIM of 0.83 ± 0.03 for an IRB-approved dataset^27^. Zhan et al. have used a latent representation-based conditional generative adversarial network to synthesize a real-like missing MRI modality using multiple modalities which already been obtained. They have performed their experiment on BraTS 2015 dataset^16^ and have got mean PSNR as 26.495 ± 3.245, normalized root-mean-square error (NRMSE) as 0.235 ± 0.09 and SSIM as 0.917 ± 0.037.

From the above discussion, we can comment that most of the previous works are based on simply training the generator to synthesize new data and the discriminator to recognize the same as real or fake. In other words, most of the works are based on one-to-one synthesis methods which attempt to synthesize a new image from the latent representation of a given source image. Since these methods are optimized for a single input channel, they can sensitively learn the unique and detailed features of the given source contrast. However, this might produce a high correlation between source and generated images^28^. Kim et al.^29^ have introduced a new architecture named **DiscoGAN**. Their proposed GAN can generate images of different domains considering the cross-domain relations effectively. Architecture-wise this model consists of two inter-domain Generators and one discriminator for each image-domains.

Basically, many-to-one synthesis methods aim to synthesize an image from a shared latent representation of multiple source images. These methods are good in capturing features that are shared across distinct source images, even when these features are weakly present in individual contrasts. Yet, a shared latent representation might also be less sensitive to complementary features that are uniquely present in a specific source image. To this end, we aim to capture both unique information of a source image and also shared information among different latent representations of multiple images. In doing so, we have proposed **AGGrGAN**, where three different GAN models are used in order to synthesize new images. We have also developed a novel aggregation method to combine these synthesized images and to get a new image. Finally, we have applied style transfer^30–33^ on the generated image using GAN, where the same is trained using the original images. After training, the style of aggregated image is transferred, where raw images are considered as style images. Therefore, by aggregation, we have considered the shared information among multiple images and we are able to capture unique features by the style transfer. We have performed our experiment on two publicly available datasets namely, BraTS 2020 dataset^16^ and brain tumor dataset^34^. The overview and main contributions of our work can be summarized as follows:

- A novel aggregation method, called **AGGrGAN**, is proposed to combine synthesized images obtained from different GAN models.
- An attempt is made to capture shared information from the latent representation of the generated images by the GAN models.
- Style transfer is performed on the aggregated image to encapsulate the localized information of the source images.
- Pixel wise aggregation of images has been performed, where the weights assigned to the images depend on what extent the corresponding pixel lies in the edge region.
- The experiments have been done on two publicly available datasets—(i) brain tumor dataset^34^, (ii) BraTS 2020 dataset^16^, and the obtained results are satisfactory when measured in terms of some standard metrics.

Overall our work consists of the following sections: (1) Introduction; (2) Results and discussion; (3) Methodology; (4) Conclusion. In the following section, we have discussed the performance of our proposed method.

## Results and discussion

**Figure 1:**
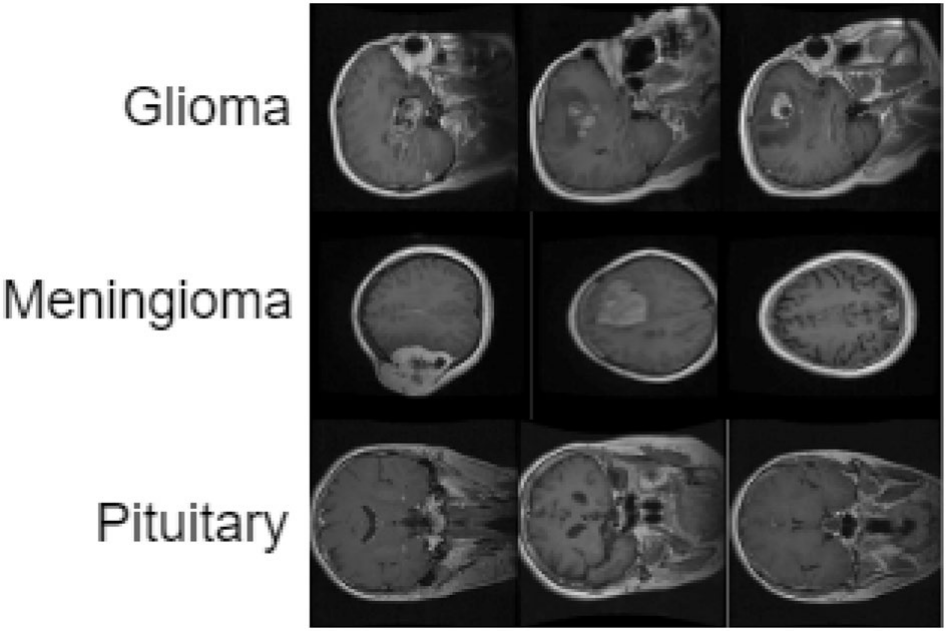
Sample images in brain tumor dataset^34^ where each row represents images of same class.

**Figure 2:**
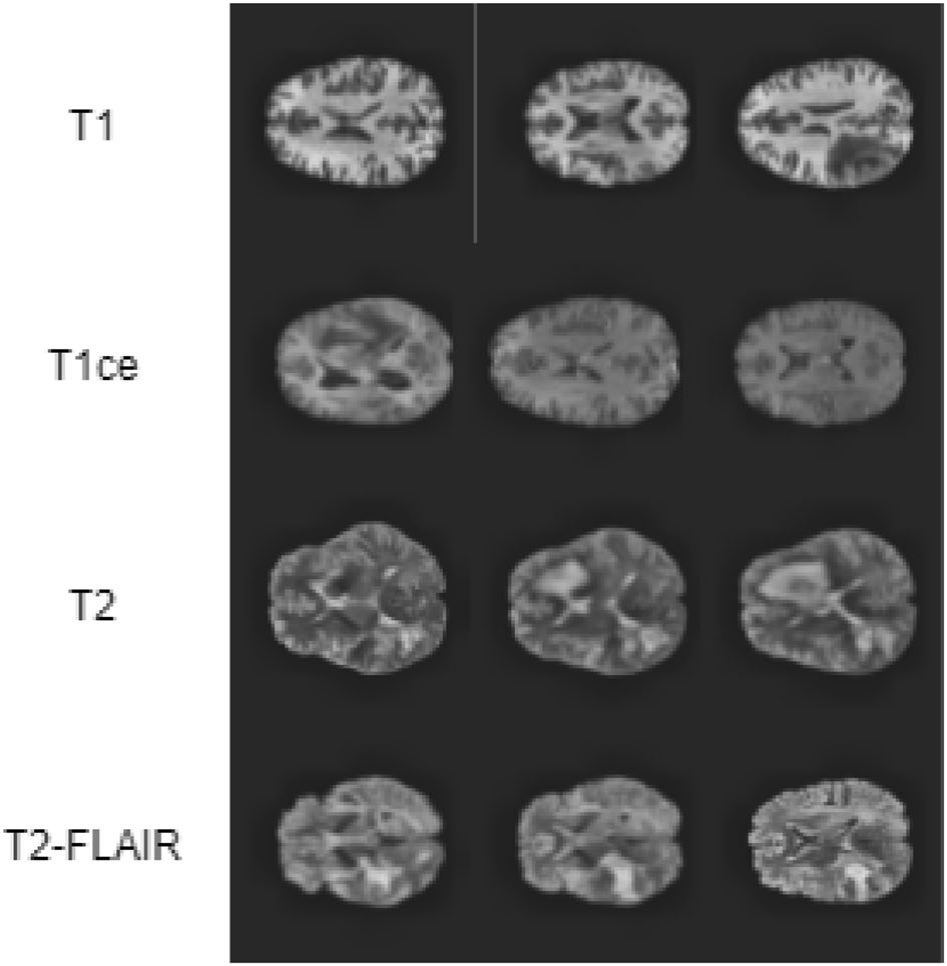
Sample images in BraTS 2020 dataset^16^ where each row represents images of the same class.

In this section, we have evaluated and have discussed our proposed method’s performance. So, we have considered the following two datasets to conduct all these evaluation-based experiments individually:

1. **Brain tumor dataset**^34^: this brain tumor dataset contains 3064 T1-weighted contrast-enhanced images with three kinds of brain tumors. The classes are given as follows:
  Glioma.
  Meningioma.
  Pituitary.
  We have shown three sample images from for each class in Fig. 1.
2. **BraTS 2020 dataset**^16^: all scans in the dataset are available as NIfTI files and the different types of data in the dataset are described below:
  Native (T1).
  Post-contrast T1-weighted (T1ce).
  T2-weighted (T2).
  T2 Fluid Attenuated Inversion Recovery (T2-FLAIR).
  These samples have has been acquired with different clinical protocols and various scanners from multiple (19) institutions. Each type contains a total of 369 NIfti files. We have extracted the images from it and created these four kinds of cases. We have a total of 1107 images for each type of data. Figure 2 shows three sample images for all the four classes present in this dataset.

To measure the performance of our proposed method over the two datasets, we have used the following four metrics to measure the performances for all three base models before applying style transfer, and after applying style transfer and **AGGrGAN**:

1. SSIM : It denotes the degree of similarity present between two images. Hence, the closure the similarity exists, the greater the SSIM index becomes whereas, two identical images have SSIM as exactly 1. SSIM is evaluated using the following formula:
  1 $$\begin{aligned} SSIM = \frac{(2\mu _x \mu _y + c_1)(2\sigma _{xy} + c_2)}{({\mu _x}^2+{\mu _y}^2+c_1)({\sigma _x}^2+{\sigma _y}^2+c_2)} \end{aligned}$$
  where, $\mu _x$, $\mu _y$ are the mean and $\sigma _x$, $\sigma _y$ are the standard deviation of the intensity values present in the two images respectively. $\sigma _{xy}$ is the covarience between the two images’ intensities. The constants $c_1$, $c_2$ are used for negating the weak denominator effect.
2. PSNR: It denotes the ratio between the maximum intensity value to the present noise value. Maximum pixel intensity is generally 255, and the square root of Mean Squared Error (MSE) between the two images can be used as an estimate of noise. Therefore, PSNR can be evaluated as-
  2 $$\begin{aligned} PSNR= & 20log_{10}\left( \frac{MAX_f}{\sqrt{MSE}}\right) \end{aligned}$$
  3 $$\begin{aligned} MSE= & \frac{1}{m \cdot n}\sum ^{m}_{i=1}\sum ^{n}_{j=1}{(f(i,j)-g(i,j))}^2 \end{aligned}$$
  where, $MAX_f$ is the maximum pixel intensity value and *f*(*i*, *j*), *g*(*i*, *j*) denote the pixel intensity value of pixel (*i*, *j*) for the two images respectively. Size of both images are $m \cdot n$. So greater value of PSNR indicates less amount of noise which means the synthetic image has closer resemblance to the original image.
3. Kullback-Leibler (KL) divergence: For any two distributions *P* and *Q*, KL divergence of *P* from *Q* can be evaluated as:
  4 $$\begin{aligned} KL(P \parallel Q) = \sum _x{P(x)\cdot log \{ P(x)/Q(x) \}} \end{aligned}$$
  where, *P*(*x*), *Q*(*x*) denote the probabilities for any event *x* occurred under *P* and *Q* respectively. Hence, more divergence between two distributions indicate higher KL divergence value. To measure the KL-divergence between two images, their corresponding histogram distribution values can be considered as *P* and *Q* whereas, every possible pixel intensity value can represent the events.
4. Sharpness Difference (SD): It indicates the degree of sharpness diversity between two images. It is evaluated based on the ratio of the square of the maximum intensity to the gradient difference ($\nabla$) between two images.
  5 $$\begin{aligned} SD= & 10log_{10}\left( \frac{{MAX_f}^2}{\nabla }\right) \end{aligned}$$
  6 $$\begin{aligned} \nabla= & \frac{1}{mn} |\Delta f(i,j)-\Delta g(i,j)| \end{aligned}$$
  7 $$\begin{aligned} \Delta f(i,j)= & 2f(i,j) - f(i-1,j) - f(i,j-1) \end{aligned}$$
  So more identical images have less gradient difference value which leads to greater SD measure.

We have considered the above-mentioned metrics for assessing the proposed model when applying on two publicly available datasets, namely brain tumor dataset^34^, and BraTS 2020 dataset^16^. For each dataset, we have recorded the metrics of the generated images considering the original images as the reference. The performances of all the categories under these two datasets have been shown in Tables 1 and 2 respectively.

**Figure 3:**
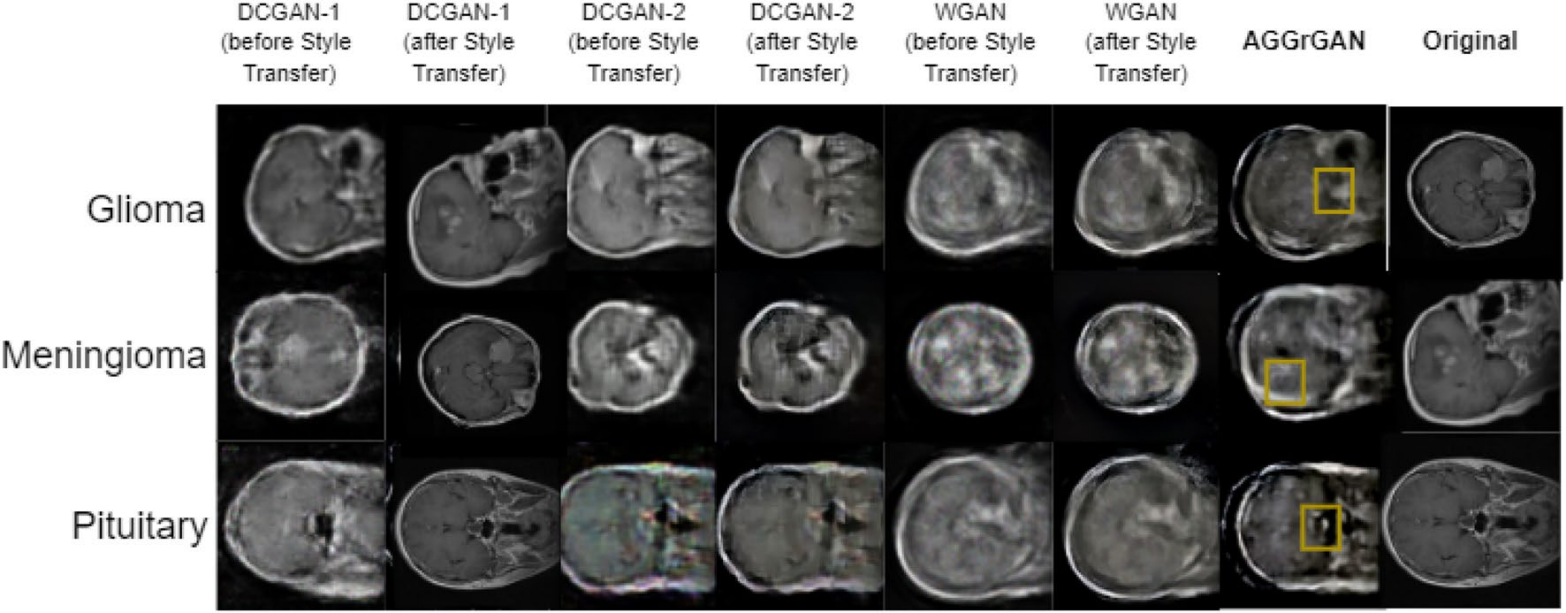
Sample generated images in the brain tumor dataset^34^ where each row represents images of the same class and each of the six leftmost columns represents images generated by the same method (tumor region produced by the **AGGrGAN** model is marked) and the rightmost column represents the original images.

First, we look into the generated images for each dataset along with the raw images so that it might be easier to understand the proposed model’s performance. Figure 3 shows all the generated images in the brain tumor dataset.

Figure 3 shows that although the generated images resemble the original images in Fig. 1, the generated images are somewhat dull compared to the original ones. However, applying style transfer may cause the resulting image to have more resemblance with the original ones. Table 1 further analyses the generated images performance-wise.

**Table 1:** Performance of the base GAN models along with **AGGrGAN** applied on the brain tumor dataset.

Category	Method	SSIM	PSNR	KL-divergence	SD
Glioma	DCGAN-1	0.39	17.33	**0.23**	28.52
	DCGAN-1+style transfer	0.56	17.80	0.37	29.68
	DCGAN-2	0.40	15.55	0.34	28.04
	DCGAN-2+style transfer	0.55	16.85	0.45	29.73
	WGAN	0.42	17.31	0.36	28.14
	WGAN+style transfer	0.52	17.65	0.45	29.48
	**AGGrGAN (SSIM)**	**0.57**	17.85	0.50	**29.78**
	**AGGrGAN (PSNR)**	0.55	**17.97**	0.44	29.50
Meningioma	DCGAN-1	0.42	16.18	**0.27**	28.97
	DCGAN-1+style transfer	0.49	16.21	0.63	29.47
	DCGAN-2	0.40	15.61	0.27	28.26
	DCGAN-2+style transfer	0.51	16.38	0.42	29.31
	WGAN	0.46	**17.92**	0.40	28.64
	WGAN+style transfer	0.48	17.59	0.48	29.09
	**AGGrGAN (SSIM)**	**0.53**	17.13	0.40	**29.61**
	**AGGrGAN (PSNR)**	0.51	17.49	0.57	29.56
Pituitary	DCGAN-1	0.37	15.35	0.38	28.54
	DCGAN-1+style transfer	0.44	15.83	0.46	28.66
	DCGAN-2	0.37	16.42	0.34	27.71
	DCGAN-2+style transfer	0.42	16.53	0.21	28.34
	WGAN	0.40	16.91	0.39	28.11
	WGAN+style transfer	0.42	16.87	0.28	28.35
	**AGGrGAN (SSIM)**	**0.47**	16.93	0.34	**28.75**
	**AGGrGAN (PSNR)**	0.44	**17.12**	**0.19**	28.54

Values in bold signify that they are—(i) the metric-wise best value obtained for the corresponding class for Tables 1, 2, 3, 4 and (ii) case-wise best performance obtained for a particular dataset.

Based on the results shown in Table 1, it is evident that our proposed **AGGrGAN (SSIM)** has generated images with the best SSIM scores for all categories of the brain tumor dataset whereas, **AGGrGAN (PSNR)** has generated images with maximum PSNR scores for Glioma and Pituitary. Our proposed method can produce images with SSIM values neighbouring 0.50. SSIM has widespread applications for measuring similarities between images in different domains, and a high SSIM score suggests a better quality of images. Although for other metrics, **AGGrGAN (SSIM)** has not delivered the best results in some cases. Especially, at least one base model with or without style transfer has better KL-divergence scores than **AGGrGAN (SSIM)** for all the three categories. Only **AGGrGAN (PSNR)** has generated the images with most KL-divergence score for pituitary. DCGAN-1 without style transfer has generated the best Glioma and Meningioma images according to KL-divergence scores. This proves that the resulting image of **AGGrGAN** may be structurally more similar to the original ones but it has a diverse histogram distribution. Overall, the addition of style transfer has improved the base models’ SSIM values for all the cases and PSNR values for most of the cases. Only WGAN has produced better Meningioma images with respect to PSNR. However, the style transferred images acquired a more diverse histogram distribution which suggests their higher KL-divergence value than that of the base models’ produced ones. style transfer has improved KL-divergence values for only WGAN in the case of Pituitary. Similar to SSIM, **AGGrGAN (SSIM)**’s generated images have the best SD results. Since the edges of the base image are prioritized during aggregation, the final output provides more edge-wise similarity thus yielding better SD results. Besides that, the style-transferred images generally have higher SD compared to their original counterparts. During style transfer, the edge characteristics of the raw images are transferred to the synthetic image as well which causes the style transferred images to have SD-wise better performance.

**Figure 4:**
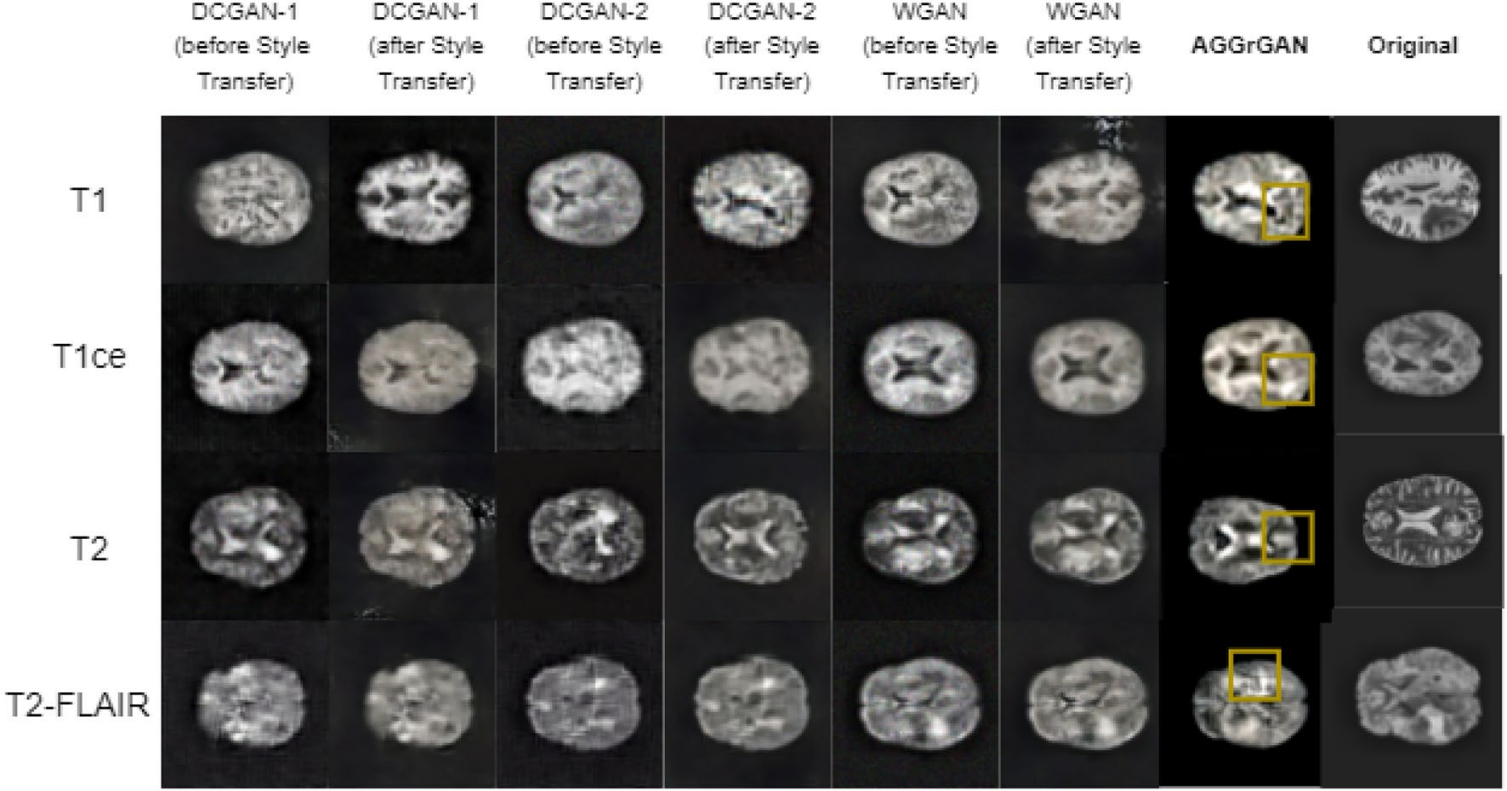
Sample generated images in the BraTS 2020 dataset^16^ where each row represents images of the same class and each of the six leftmost columns represents images generated by the same method (tumor region produced by the **AGGrGAN** model is marked) and the rightmost column represents the original images.

We can observe the synthetic images for the BraTS 2020 dataset in Fig. 4 which shows that the generated images are more clearer compared to that of the brain tumor dataset.

To further analyze the images present in Fig. 4, Table 2 has been provided with the metric-wise performance.

**Table 2:** Performance of the base GAN models along with **AGGrGAN** applied on the BraTS 2020 dataset.

Category	Method	SSIM	PSNR	KL-divergence	SD
T1	DCGAN-1	0.47	17.9	5.05	28.52
	DCGAN-1+style transfer	0.69	19.69	1.71	28.91
	DCGAN-2	0.68	19.69	3.83	27.70
	DCGAN-2+style transfer	0.76	20.61	1.17	29.05
	WGAN	0.62	20.62	2.37	27.51
	WGAN+style transfer	0.76	21.07	1.34	28.99
	**AGGrGAN (SSIM)**	**0.77**	21.14	1.16	**29.14**
	**AGGrGAN (PSNR)**	0.74	**22.12**	**1.10**	29.10
T1ce	DCGAN-1	0.48	18.60	3.93	28.51
	DCGAN-1+style transfer	0.72	21.53	1.76	28.96
	DCGAN-2	0.65	19.70	1.53	27.57
	DCGAN-2+style transfer	0.79	22.10	1.23	28.81
	WGAN	0.61	20.20	2.37	27.52
	WGAN+style transfer	0.82	22.85	1.19	29.12
	**AGGrGAN (SSIM)**	**0.83**	23.30	1.11	**29.15**
	**AGGrGAN (PSNR)**	0.82	**23.68**	**0.99**	29.07
T2	DCGAN-1	0.49	18.76	3.70	28.41
	DCGAN-1+style transfer	0.68	19.71	1.92	28.86
	DCGAN-2	0.69	19.67	3.54	27.82
	DCGAN-2+style transfer	0.77	20.63	1.21	29.55
	WGAN	0.60	21.14	2.40	27.46
	WGAN+style transfer	0.77	21.28	1.25	29.10
	**AGGrGAN (SSIM)**	**0.80**	21.60	1.20	**29.63**
	**AGGrGAN (PSNR)**	0.78	**21.94**	**1.15**	29.11
T2-FLAIR	DCGAN-1	0.48	17.81	1.65	29.05
	DCGAN-1+style transfer	0.65	19.05	1.34	28.54
	DCGAN-2	0.65	21.27	1.48	27.62
	DCGAN-2+style transfer	0.76	21.33	1.27	28.93
	WGAN	0.54	19.91	2.06	27.49
	WGAN+style transfer	0.77	21.04	1.32	29
	**AGGrGAN (SSIM)**	**0.78**	**21.90**	1.26	29.11
	**AGGrGAN (PSNR)**	0.77	21.77	**1.18**	**29.35**

Values in bold signify that they are—(i) the metric-wise best value obtained for the corresponding class for Tables 1, 2, 3, 4 and (ii) case-wise best performance obtained for a particular dataset.

Table 2 shows that the results are far better in the BraTS 2020 dataset. The proposed **AGGrGAN** model (considering both SSIM and PSNR based aggregation) has outperformed all the base models in the case of all the performance metrics. PSNR based **AGGrGAN** has the best KL-divergence score results for all the classes. One point is to be noted that the performance of DCGAN-1 is poorer than the other two. AGGrGAN takes advantage of choosing the best two models and further applies logical aggregation technique so it is not affected by DCGAN-1’s poor performance and further improves from the other two by selecting their best features only. Another reason could be attributed to the fact that DCGAN-2 and WGAN have more or less the same performance which is not that much is reflected in the results shown in Table 1. High SSIM values around 0.80 suggest that the generating images are more resembling the original ones which further ensures the success of the GAN. The only drawback has occurred for the KL-divergence scores as all the scores exceed 1 which means that diversity in histogram remains at a larger scale. To conclude, **AGGrGAN**, we can say that base GAN models have performed better on the BraTS 2020 dataset than that on the brain tumor dataset.

**Figure 5:**
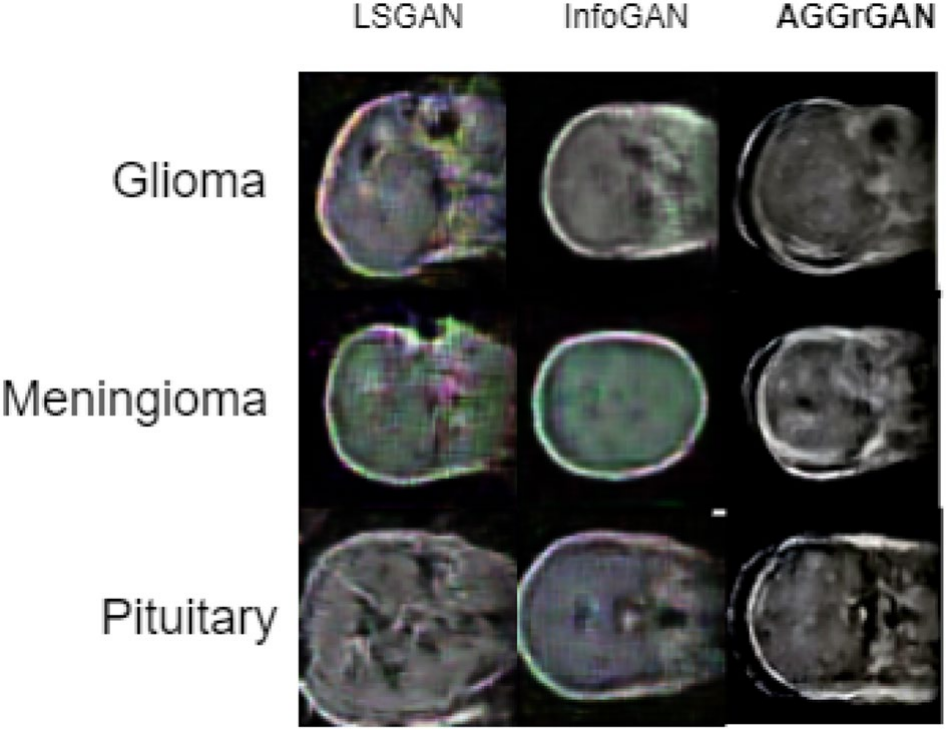
Visual comparison between samples images generated by existing GAN architectures like LSGAN, InfoGAN and the proposed **AGGrGAN** in brain tumor dataset^34^ where each row represents images of the same class and each column represents images generated by the same method.

Next, we analyze our proposed **AGGrGAN**’s performance in a competitive scenario by comparing with two basic GAN models: (i) Least Squares GAN (LSGAN)^35^, (ii) Information maximizing GAN (InfoGAN)^36^. In this scenario, we have considered SSIM based **AGGrGAN** only. First, a side-by-side comparison of the images generated by these two models and **AGGrGAN** for the brain tumor dataset^34^ is shown in Fig. 5.

Figure 5 shows that the images generated by LSGAN and InfoGAN are more faded compared to that of **AGGrGAN** which indicates the upper hand in our proposed architecture’s performance. We have also provided the metric-wise performance in Table 3.

**Table 3:** Quantitative comparison of performance between existing GAN architectures like LSGAN, InfoGAN and the proposed **AGGrGAN** applied on the brain tumor dataset.

Category	Method	SSIM	PSNR	KL-divergence	SD
Glioma	LSGAN	0.48	17.52	**0.26**	28.57
	InfoGAN	0.43	17.22	0.28	28.63
	**AGGrGAN**	**0.57**	**17.85**	0.50	**29.78**
Meningioma	LSGAN	0.45	**17.96**	**0.25**	28.75
	InfoGAN	0.43	16.48	0.31	28.18
	**AGGrGAN**	**0.53**	17.13	0.40	**29.61**
Pituitary	LSGAN	0.38	16.61	0.31	27.68
	InfoGAN	0.44	16.75	**0.24**	27.80
	**AGGrGAN**	**0.47**	**16.93**	0.34	**28.75**

Values in bold signify that they are—(i) the metric-wise best value obtained for the corresponding class for Tables 1, 2, 3, 4 and (ii) case-wise best performance obtained for a particular dataset.

**Figure 6:**
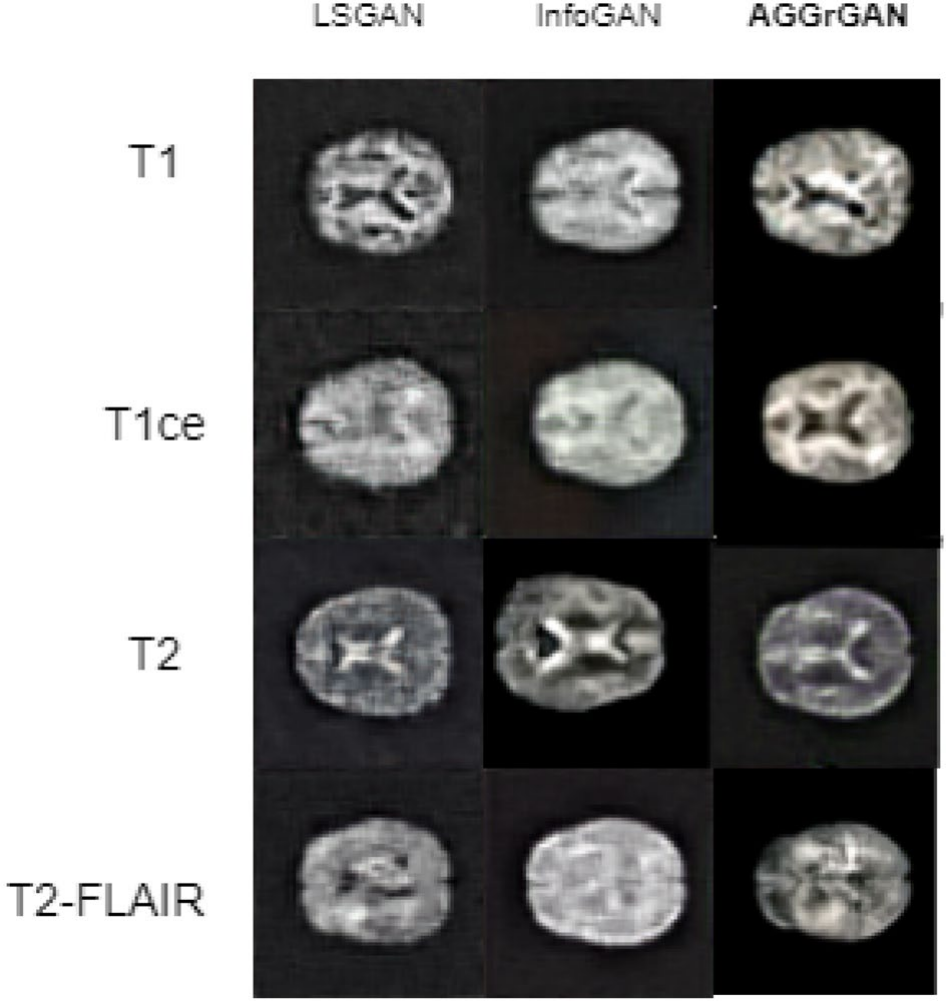
Visual comparison between sample generated images by existing GAN architectures like LSGAN, InfoGAN and the proposed **AGGrGAN** in the BraTS 2020 dataset^16^ where each row represents images of the same class and each column represents images generated by the same method.

Table 3 shows us that **AGGrGAN** has higher SSIM and SD values compared to the other two which justifies the success of edge-based priority-based aggregation of base architecture outputs. **AGGrGAN** has also outperformed the other models with respect to PSNR for the classes Glioma and Pituitary. Meanwhile, for the Meningioma class, InfoGAN has generated the best images with response to PSNR values. However, **AGGrGAN** has not the best results for the KL-divergence score. LSGAN has the lowest KL-divergence scores for Glioma and Pituitary whereas, InfoGAN has for the Meningioma class. Next, this kind of comparison is provided for the BraTS 2020 dataset^16^. Figure 6 shows the images generated by LSGAN, InfoGAN and **AGGrGAN**.

The synthetic images generated by LSGAN, InfoGAN and **AGGrGAN** are almost similar. All of these images are appealing and bear a strong resemblance to the original ones. So we have further provided the performance metric wise comparison in Table 4.

**Table 4:** Quantitative comparison of performance between existing GAN architectures like LSGAN, InfoGAN and the proposed **AGGrGAN** applied on the the BraTS 2020 dataset.

Category	Method	SSIM	PSNR	KL-divergence	SD
T1	LSGAN	0.69	20.17	**1.14**	27.53
	InfoGAN	0.76	**21.35**	3.55	27.82
	**AGGrGAN**	**0.77**	21.14	1.16	**29.14**
T1ce	LSGAN	0.58	20.31	1.52	27.49
	InfoGAN	0.72	19.51	1.87	27.64
	**AGGrGAN**	**0.83**	**23.30**	**1.11**	**29.15**
T2	LSGAN	0.68	20.16	1.39	27.60
	InfoGAN	0.75	21.56	2.63	27.75
	**AGGrGAN**	**0.80**	**21.60**	**1.20**	**29.63**
T2-FLAIR	LSGAN	0.63	19.91	1.49	27.49
	InfoGAN	0.68	17.47	2.08	27.76
	**AGGrGAN**	**0.78**	**21.90**	**1.26**	**29.11**

Values in bold signify that they are—(i) the metric-wise best value obtained for the corresponding class for Tables 1, 2, 3, 4 and (ii) case-wise best performance obtained for a particular dataset.

Table 4 shows that **AGGrGAN** has the best outcome for most of the cases. It has provided the best results with respect to all of the metrics for T1ce, T2 and T2-FLAIR. Only cases where our proposed approach is outperformed by any other model are PSNR and KL-divergence for T1. Overall, beating other models in most cases ensures the robustness and efficiency present in our proposed approach.

We have also used standard CNN models, namely InceptionResNetV2^37^, VGG19^38^ and ResNet152V2^26^ to perform classification based evaluation of the images generated by our proposed model. We have generated 300 images for each class for both datasets. To measure the performance for the generated images w.r.t. the original images, we have performed such classification for two different cases, which are: (i) **Case-1:** considering the original images and the generated images as training and test sets respectively, (ii) **Case-2:** considering 80% of the mixture of original and generated images as training data and the remaining as testing data. Furthermore, we have experimented with whether the addition of generated images to the training dataset causes any improvement of the performance and whether our proposed method can beat traditional data augmented methods such as flip, shift and zoom. To conduct such experiments, three additional test cases have been considered, which are—(i) **Case-3(a):** the raw image dataset has been divided into training and test sets by 80:20 ratio. (ii) **Case-3(b): ** instead of using 80% of the raw images only as training data, we have also included all the generated images in the training data. (iii) **Case-3(c): ** we have created 100 images using each of the three images augmented methods- flip, shift and zoom to achieve 300 augmented images per class and have added to the 80% of the raw images for training data. For each case, we have used 20% of the training data for validation purpose. Each CNN model is trained for 20 epochs and a batch size of 32 is considered. Since the brain tumor dataset^34^ is a class-imbalanced dataset, we have considered an equal number of images for each class. The results of these experiments are noted in Table 5.

**Table 5:** Classification performance of generated images in brain tumor dataset and BraTS 2020 dataset, where **Case-1:** considering the complete original images and the generated images as training and testing data respectively, **Case-2:** considering 80% of the mixture of original and generated images as training data and the remaining as testing data and **Case-3:** considering 20% of the original images as testing data and choosing the training data by three different ways: (a) remaining 80% only, (b) remaining 80% along with all generated images and (c) remaining 80% along with augmented images created by- shift, flip and zoom.

Dataset	Case	Accuracy (%)—InceptionResNetV2	Accuracy (%)—VGG19	Accuracy (%)—ResNet152V2
Brain tumor dataset	Case-1	44.43	**77.12**	58.45
	Case-2	78.15	**81.23**	78.56
	Case-3 (a)	87.29	90.12	**92.24**
	Case-3 (b)	90.35	92.70	**93.88**
	Case-3 (c)	90.11	**93.41**	92.47
BraTS 2020 dataset	Case-1	59.05	**61.79**	53.12
	Case-2	86.17	**88.28**	85.65
	Case-3 (a)	88.57	**91.27**	88.18
	Case-3 (b)	89.72	**94.24**	92.80
	Case-3 (c)	90.20	**93.66**	92.03

Table 5 shows that the performance is more diverse in Case-1 compared to Case-2. In Case-2, the pairwise accuracy difference does not exceed 4%. Overall, VGG-19 provides accuracy-wise better results compared to the other two CNN models for both cases. Based on the case-wise results, it can be said that the Case-2 shows better accuracy values for all three CNN models considered here than Case-1. In the Case-2, each model predicts with accuracy greater than 78% and 85% for brain tumor dataset and BraTS 2020 dataset respectively, whereas only VGG-19 provides more than 75% accurate results for the brain tumor dataset in the Case-1. We can also observe that each model has performed better for Case-3(b) than Case-3(a) by at least 2% higher accuracy for most of the cases. Thus, the goodness of the generated images can be ensured. Based on the comparison between two cases i.e., Case-3(b) and Case-3(c), we can see that Case-3(b) outperforms Case-3(c) for almost all the scenarios. Only exceptions are—(i) VGG19-brain tumor dataset and (ii) InceptionResNetV2—BraTS 2020 dataset. Such comparison proves the effectiveness of **AGGrGAN** with respect to traditional image augmentation procedures. Also, all these cases provide high accuracy. Hence, all these results can be considered as satisfactory keeping in mind the complexity of brain tumor images.

## Methodology

In this section, we have mainly discussed our proposed method along with its detailed architecture. We have also described the benchmark datasets briefly. This section consists of mainly two subsections: (i) Datasets Used, (ii) Proposed method and **AGGrGAN** Architecture.

### Proposed method and AggrGAN architecture

In the present work, we have proposed a novel GAN model, called **AggrGAN**, where images generated by the different variants of GAN are aggregated. Finally, we have applied *style transfer* on the aggregated image. Before we move deeper into the architecture, we will briefly discuss GANs, DCGAN, WGAN, Aggregation method and style transfer.

#### Generative adversarial networks

GANs have proved to be very effective for image generation in different computer vision tasks^39^ since the breakthrough work by Goodfellow et al.^13^. GANs have shown promise in generating highly realistic images without a well-defined objective function and also the generator of GAN can learn from extremely small varieties in data. GAN is a deep learning architecture that consists of two models—a generative model G and a discriminative model D. The generative model captures the data distribution. The discriminative model estimates the probability for the sample that if it is drawn from the training data rather than the generative model. The two models are simultaneously trained via an adversarial process. The architecture follows a game theory approach, and it corresponds to a minimax two-player game^13^. The training procedure of G is to maximize the probability of D making a mistake.

**Figure 7:**
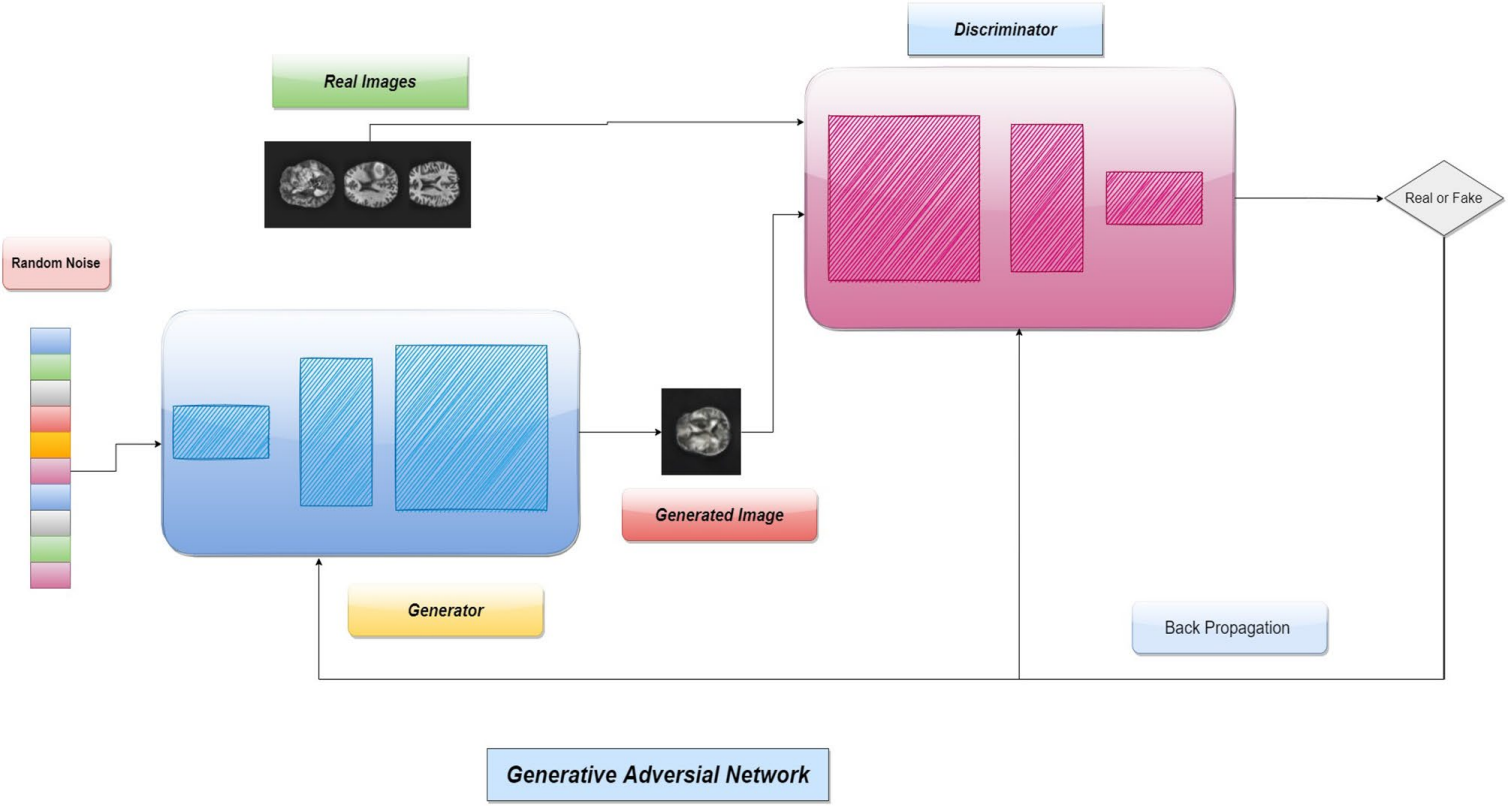
A workflow of generative adversarial network (sample real image source: Br 2020 dataset^16^).

Let the generator G (z, ${\theta }_x$) is a differentiable function represented by a multilayer perceptron with parameters ${\theta }_g$ that depicts a mapping to the data space. To learn the generator’s distribution $\rho _g$ over the data space x, a prior $\rho _z$ is defined on the random input noise variable z. The discriminator D (x, ${\theta }_d$) is also a neural network that gets a sample from the real dataset or the generated synthetic dataset produced by G and outputs a single scalar value that the input data comes from the real training dataset. The training process focuses on the task that the discriminator D will maximize the probability of assigning correct labels to the training examples and generated samples from G. At the same time, G is trained to generate data samples similar to the real dataset so that D cannot differentiate them from actual data. It is formulated as a minimax two-player game with value function V (G, D), as defined in Eq. (8):

8 $$\begin{aligned} \underset{G}{min} \underset{D}{max} V(D, G) = E_{x \sim \rho _{data}(x)}[log D(x)] + E_{z\sim \rho _{data}(z)} [log(1-D{(x)})] \end{aligned}$$

where x is the real data and z is the input random noise. $\rho _{data}$, $\rho _z$ represent the distribution of the real data and the input noise respectively. This can be reformulated as the minimization of the Jensen-Shannon (JS) divergence between the distribution $\rho _{data}$ and another distribution $\rho _g$ derived from $\rho _z$ and G. D(x) represents the probability that x came from the real data while G(z) represents the mapping to synthesize the real data. The generator G is a deeper neural network and has more convolutional layers and non-linearities. The noise vector z is upsampled while G learns the weights through backpropagation. At some point, the generator starts producing data that are classified as real by the discriminator. A workflow of GANs is given in Fig. 7.

#### Deep convolutional generative adversarial network

DCGAN^18^ is a major improvement on the first GANs^13^. DCGAN can generate better quality images and has more stability during the training stage. In the synthetic image generation process using the DCGAN, there are two phases: a learning phase and a generation phase. In the training phase, the generator draws samples from an N-dimension normal distribution and works on this random input noise vector by successive upsampling operations, eventually generating an image from it. The discriminator attempts to distinguish between images drawn from the generator and images from the training set^18^. Two important features of DCGAN are BatchNorm^40^ for regulating the extracted feature scale, and LeakyRelu^41^ for preventing dead gradients. DCGAN also replaces all max pooling with a convolutional stride and uses transposed convolution for upsampling. It eliminates fully connected layers and uses batch normalization. DCGAN uses Rectified Linear Unit (ReLU) in the generator except for the output which uses Tanh and uses LeakyReLU in the discriminator.

#### Wasserstein generative adversarial network

WGAN^20^ is an alternative to traditional GAN which improves the stability during training of the model and provides a loss function that correlates with the quality of generated images. The development of the WGAN has a strong mathematical base, although in practice it requires only a few minor modifications to the established standard DCGAN. In WGAN, instead of using a discriminator to classify or predict the probability of generated images as being real or fake, it changes or replaces the discriminator model with a critic that scores the realness or fakeness of a given image. This change is motivated by a mathematical argument that training the generator should seek a minimization of the distance between the distribution of the data observed in the training dataset and the distribution observed in generated examples. The argument contrasts different distribution distance measures, such as KL divergence, JS divergence, and the Earth-Mover (EM) distance (also known as the Wasserstein-1 metric) and given in Eq. (9):

9 $$\begin{aligned} W(p_g, p_r) = \underset{{\gamma \in \prod (p_g,p_r)}}{inf} E_{{(x,x^{\prime })}\sim \gamma } ||{x - {x^{\prime }}}|| \end{aligned}$$

where $\prod$($p_g$, $p_r$) is the set of all joint distributions $\gamma (x,x^\prime )$ whose marginals are $p_g$ and $p_r$, respectively. In other words, p implies how much mass must be transported from one distribution ($p_r$) to another ($p_g$). This distance intuitively indicates the cost of the optimal transport plan^20^.

In our work, we have used Wasserstein Divergence GAN^21^ as Wasserstein-1 metric cannot be directly applied as an objective without imposing the strict k-Lipschitz constraint^21,42^. The objective function is given as in Eq. (10).

10 $$\begin{aligned} L_{DIV} = {\underset{{x \sim p_r}}{E}[f(x)] } - {\underset{{\hat{x} \sim p_g}}{E}[f(\hat{x})]} + k{\underset{{\hat{x} \sim p_u}}{E}[||\nabla f(\hat{x})||^p] } \end{aligned}$$

Here $p_g$ and $p_r$ have same meaning as in Eq. (9). $p_u$ is a Radon probability^43^ measure, $\nabla$ is the gradient operator, $p > 0$. So the optimization problem can be formulated as given in Eq. (11).

11 $$\begin{aligned} \underset{G}{min} \underset{D}{max} V(D,G) = {\underset{{G(z) \sim p_g}}{E}[D(G(z))] } - {\underset{{x \sim p_r}}{E}[f([D(x))]} + k{\underset{{\hat{x} \sim p_u}}{E}[||{\nabla }_{\hat{x}} f(D(\hat{x}))||^p] } \end{aligned}$$

where *z* is random noise, *x* is the real data, and $\hat{x}$ is sampled as a linear combination of real and fake data points.

In our research, we have used three GANs i.e., one WGAN and two variants of DCGAN which are different in terms of their upsampling method in the Generator. After we obtain the generated image from the different GANs, we aggregate them by our proposed aggregation method.

#### Aggregation method

We have selected two images out of the three images with closer metric scores (i.e., SSIM or PSNR value) generated by the three GAN models according to their metric value assessed w.r.t. the raw images (i.e., top-two images are selected based on the metric score). Then, we have aggregated the two selected images in the following manner:

- We apply Sobel^44^ filters on both images and generate the corresponding edge-mapped images.
- After this, we find the Gaussian^45^ value of the intensities of edge-mapped image as $g_1(x,y)$ and $g_2(x,y)$ respectively, where $g_i(x,y)$= Gaussian-Function(i(x,y)) of edge mapped image i).
- Then we assign weights to each edge-mapped image based on the Gaussian value for each pixel. The weight assignment method is described in Algorithm 1. Here, we have generalized the approach for different metrics that means the aggregation can be done based on the SSIM or PSNR value of each image.
- We finally aggregate the selected two generated images pixel-wise based on these weights.

**Figure 8:**
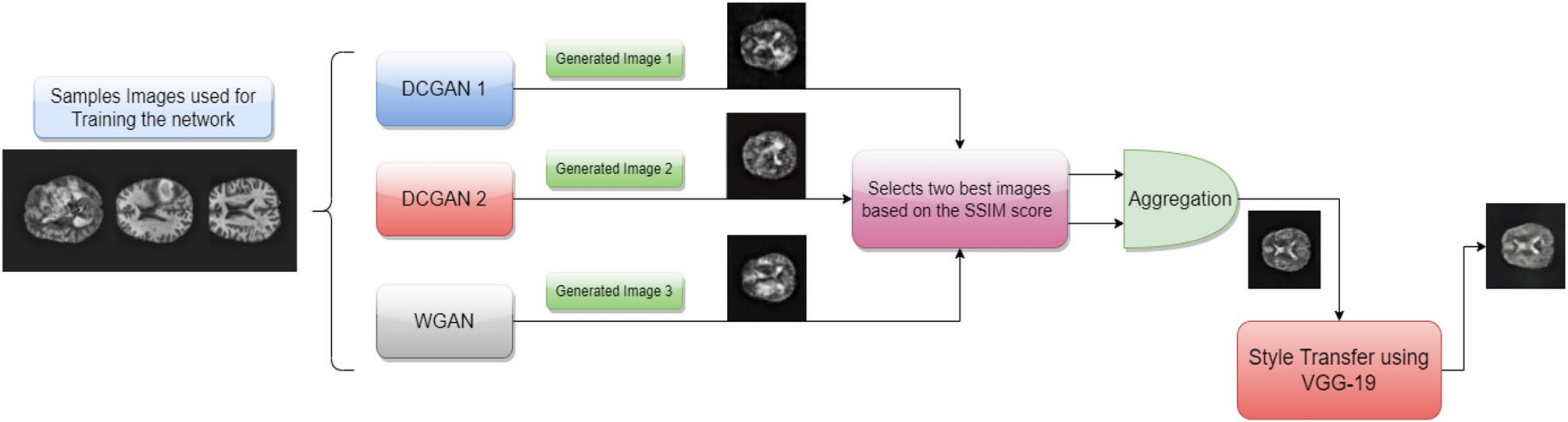
The working procedure of the proposed aggregation method (sample image source: BraTS 2020 dataset^16^).

This aggregated image will further be processed using the style transfer method. The overall working procedure of the aggregation method is given in Fig. 8.

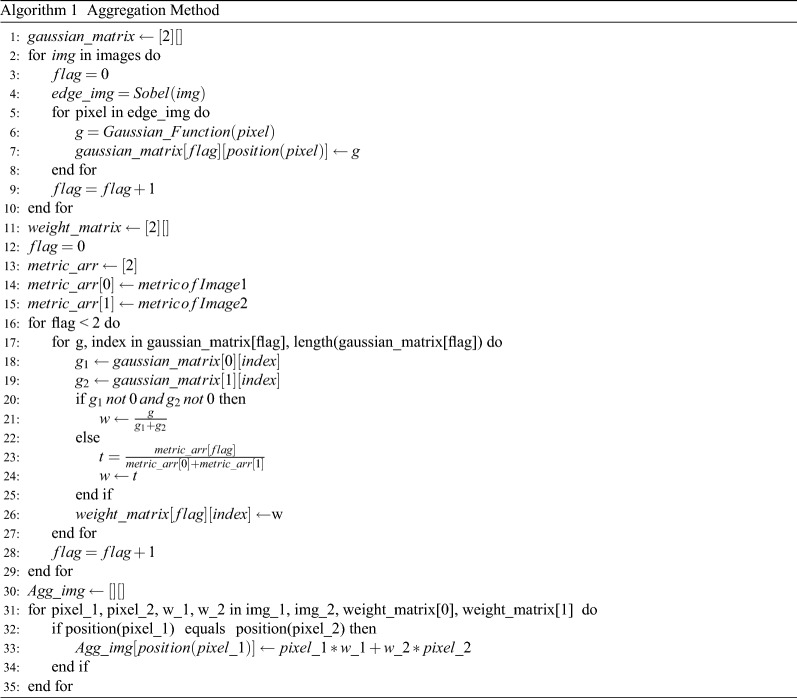



#### Style transfer

Style transfer^30–32^ is applied to generate the image, of which style is equivalent to the style of the raw image, and the content is equal to the content of the aggregated image, obtained by using the proposed aggregation method. To define a style and a content representation clearly, a loss function can be used, which shows how far away our synthesized images are from the perfect style transfer. Without style transfer, the synthetic images from the generator are rather smooth, hence style transfer is applied to make the synthetic images more similar to real ones. We hold the idea of style transfer and employ the popular CNN model, called VGG-19^38^, to extract content features and style features from multiple convolutional layers. Next, we need to understand the losses calculated during each iteration of style transfer. These are further back-propagated throughout the whole network to update all the weights.

1. ***Content Loss*** Given the chosen content layer *l*, the content loss is defined as the Euclidean distance between the feature map $F^l$ of our content image *x* and the feature map $P^l$ of our generated image $\widehat{x}$. Suppose, $F^{l}_{i,j}$ and $P^{l}_{i,j}$ are the feature values of the *i*-th filter at position *j* in layer *l* for the images *x* and $\widehat{x}$, then the content loss $L_{cont}$ can be defined as follows:
  12 $$\begin{aligned} L_{cont} = \sum _{l=0}\sum _{i,j}\frac{1}{2}({F^{l}_{i,j}}-{P^{l}_{i,j}})^2 \end{aligned}$$
2. ***Style Loss*** For each style layer, we find the pairwise correlation between all the filters’ feature vectors so that we can maintain a resemblance between the style image and the output image based on the spatial information. These feature correlations are given by Gram matrix ${G^{/}_{i_1,i_2}}$, where ${G^{/}_{i_1,i_2}}$ denotes the inner product between the vectorized feature map of filters $i_1$ and $i_2$ in layer l:
  13 $$\begin{aligned} {G^{l}_{i_1,i_2}} = \sum _{k}^{F^{l}_{i_1,k}}{F^{l}_{i_2,k}} \end{aligned}$$
  Suppose there are total $A_l$ filters each having the feature map of size $B_l$ and we have the Gram matrices $G^{l}_{i_1,i_2}$, $H^{l}_{i_1,i_2}$ for the style image *y* and the output image $\widehat{x}$. So we can evaluate the total Style Loss $L_{sty}$ as:
  14 $$\begin{aligned} L_{sty} = \sum _{l}w_l\cdot \frac{1}{4{A_l}^2{B_l}^2}\sum _{i_1,i_2}{(G^{l}_{i_1,i_2}-H^{l}_{i_1,i_2})}^2 \end{aligned}$$
  where, $w_l$ is the weight assigned to layer *l*. In this case, each $w_l$ holds the value $\frac{1}{Total\,number\,of\,style\,layers}$ i.e. $\frac{1}{5}$.
3. ***Total Variation Loss*** Furthermore, by combining the following total variation losses ($\overline{x}$ for generated phantoms, $\overline{x} \in R^{W*H}$ ), we can perform spatial smoothing in the synthesized brain tumor images.
  15 $$\begin{aligned} L_{tv} = \sum _{w,h}^(||{\overline{x}_{w,h+1}} - {\overline{x}_{w,h}} ||^2_2+|| {\overline{x}_{w+1,h}} - {\overline{x}_{w,h}}||^2_2) \end{aligned}$$
  the image size of w, h $\in$ W, H, and $x_{w,h}$ denotes the pixel value of the given position in the generated image $\overline{x}$.
  16 $$\begin{aligned} L_{ST} = w_{cont}L_{cont} + w_{sty}L_{sty} + w_{tv}L_{tv} \end{aligned}$$
  The total loss value of the style transfer network covers content loss, style loss, as well as variation loss, where $w_{cont}, w_{sty}, w_{tv}$ denote the weight of $L_{cont}, L_{sty}, L_{tv}$, respectively. So, in our research, we will optimize this total variation loss $L_{ST}$ and generate images more similar to raw images.

#### Architecture of the proposed AggrGAN model

Our architecture consists of three GANs and one style transfer model, which uses CNN to do the operation, and the aggregation method which is described in Algorithm 1. In this section, we have described the architectural details of all the models that have been used.

#### DCGAN architecture

The two variants of DCGAN are described here:

1. **DCGAN-1:** The first variant has architecture similar to the DCGAN architecture reported in^18^. We have used LeakyRelu^41^ in each layer for both generator and discriminator (except the last layer). We have used dropout of 0.25 in each layer of discriminator except the last layer, where we have used a fully connected layer with a sigmoid^46^ activation function. All the convolution layers are strided instead of pooling layer and the filter size is 4 * 4 in both the generator and the discriminator. The batch size of 64, learning rate of 0.0002 with Adam optimizer^47^ and total 1000 epochs have been used for the network.
2. **DCGAN-2:** The second variant has also similar network to the DCGAN architecture reported in^18^. The major difference of this with the first variant is that we have used transpose convolutional layer^48^ in the generator instead of using traditional convolution layer and upsampling method. It performs both the upsample operation and interprets the coarse input data to fill in the detail during upsampling. The discriminator uses traditional convolution layer and both convolution layers in the generator and the discriminator are strided, and all the filters are of size 4 * 4. The batch size of 128, learning rate of 0.0002 with Adam optimizer^47^ and total 650 epochs have been used for this case.

The main difference in these variants is how the upsampling is done in the generator of the GANs.

#### WGAN architecture

The architecture of WGAN is the same as given in^21^. We have a used fully connected layer in both generator and the discriminator, and LeakyRelu^41^ as an activation function. We have used Adam optimizer^47^ with a learning rate of 0.001 and the number of epochs used for training is 1000. We have used a mini-batch of the size of 32 in this network.

#### Architecture of style transfer

**Figure 9:**
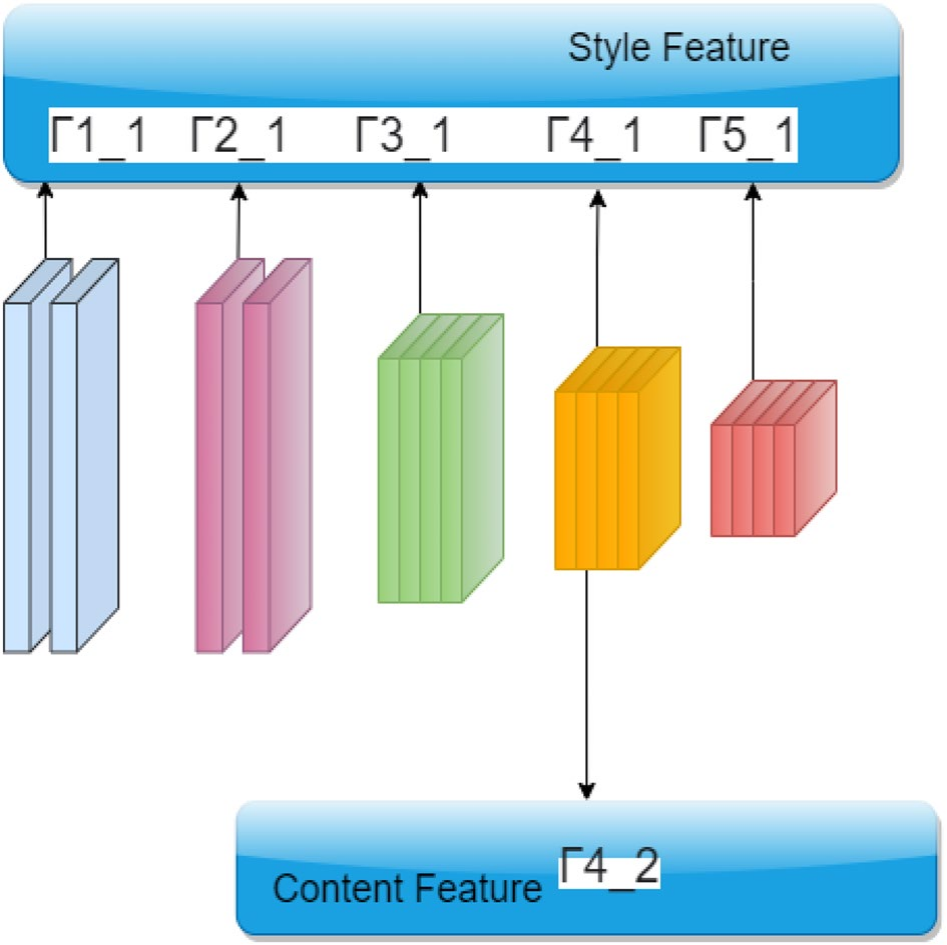
Architecture of VGG-19 network used in style transfer where the layers $\Gamma 1 (1), \Gamma 2 (1)$.. capture style features and the layer $\Gamma 4(2)$ captures the content features.

Finally we have applied style transfer on the aggregated image obtained via Algorithm 1. The VGG-19 network extracts the style of a style image and the content of a content image for mixing to generate the final output. Some layers in the network structure are adopted to extract style and content features, as shown in Fig. 9, the style index set is $\Gamma _s$ = 1_1, 2_1, 3_1, 4_1, 5_1 and the content index $\Gamma _c$ = 4_2 where index $i\_j$ denotes the *j*-th layer of the *i*-th sub-network in the overall VGG-19 architecture. Each of the style and content layers is introduced with ReLU activation function. Besides, there are average pooling layers sittuation in between the Convolution layers. The different layer structures of the VGG-19 network can be better understood from Fig. 9. The weights of the three corresponding loss functions are expressed as $w_{cont}, w_{sty}, w_{tv}$ in Eq. (16) with coefficients of 5, 100 and 0.001 respectively. The Limited memory Broyden–Fletcher–Goldfarb–Shanno (L-BFGS)^49^ optimizer is used here and total 2000 iterations are used for training the network.

## Conclusion

Information received from a small number of available medical images might be inadequate for proper medical analysis using deep learning-based approaches. Classifiers that need a vast amount of data, might suffer the most for performing classification or segmentation on a small-sized dataset. Thus to resolve this issue, synthetic images, which are consistent with the original medical images are required as an addition to the original data. This implies that synthetic medical image generation holds a promising future and has a scope of opening multiple possibilities in the medical imaging domain. In addition to that such approaches help to reduce the cost required to prepare the original medical data. In this work, we have proposed a new model for synthetic medical data generation called AGGrGAN. It uses the advantage of selecting distributed features across the multiple latent representations and captures the local patterns as well. Since our base models are already well-known and robust, the aggregation of the best two among them is ensured to bring good results than a stand-alone GAN model. However, our proposed aggregation method is limited to combining two images which can be further extended. In the future, we also plan to apply this model to other medical imaging domains where the number of sample images is scarce.

## Data Availability

The brain tumor dataset analysed during the current study are available in the Figshare repository, https://figshare.com/articles/dataset/brain_tumor_dataset/1512427 BRATS 2020 dataset analysed during the current study are available in the Multimodal Brain Tumor Segmentation Challenge 2020: Data repository, https://www.med.upenn.edu/cbica/brats2020/data.html For the generated images, we did not aim to create a dataset and we generated the images to check the performance of our method.

## References

[1] Islam J, Zhang Y (2020). Gan-based synthetic brain pet image generation. Brain Inform..

[2] Vargo M (2011). Brain tumor rehabilitation. Am. J. Phys. Med. Rehabil..

[3] Sizoo EM (2010). Symptoms and problems in the end-of-life phase of high-grade glioma patients. Neuro Oncol..

[4] Havaei M (2017). Brain tumor segmentation with deep neural networks. Med. Image Anal..

[5] Pereira S, Pinto A, Alves V, Silva CA (2016). Brain tumor segmentation using convolutional neural networks in MRI images. IEEE Trans. Med. Imag..

[6] Chen J (2016). Alu methylation serves as a biomarker for non-invasive diagnosis of glioma. Oncotarget.

[7] Liu J (2014). A survey of MRI-based brain tumor segmentation methods. Tsinghua Sci. Technol..

[8] Shivhare SN, Kumar N, Singh N (2019). A hybrid of active contour model and convex hull for automated brain tumor segmentation in multimodal MRI. Multimedia Tools Appl..

[9] Liu J (2020). Iouc-3dsfcnn: segmentation of brain tumors via IoU constraint 3D symmetric full convolution network with multimodal auto-context. Sci. Rep..

[10] Dar SU (2019). Image synthesis in multi-contrast MRI with conditional generative adversarial networks. IEEE Trans. Med. Imag..

[11] Shin H-C (2016). Deep convolutional neural networks for computer-aided detection: Cnn architectures, dataset characteristics and transfer learning. IEEE Trans. Med. Imag..

[12] Shin, H.-C., et al. Medical image synthesis for data augmentation and anonymization using generative adversarial networks. In: *International workshop on simulation and synthesis in medical imaging*, pp. 1–11 (Springer, New York, 2018).

[13] Goodfellow, I. J., et al. Generative adversarial networks. arXiv preprint arXiv:1406.2661 (2014).

[14] Li Q, Yu Z, Wang Y, Zheng H (2020). Tumorgan: a multi-modal data augmentation framework for brain tumor segmentation. Sensors.

[15] Petersen RC (2010). Alzheimers disease neuroimaging initiative (adni): clinical characterization. Neurology.

[16] Menze BH (2014). The multimodal brain tumor image segmentation benchmark (brats). IEEE Trans. Med. Imag..

[17] Isola, P., Zhu, J.-Y., Zhou, T. & Efros, A. A. Image-to-image translation with conditional adversarial networks. In: Proceedings of the IEEE conference on computer vision and pattern recognition, pp. 1125–1134 (2017).

[18] Radford, A., Metz, L. & Chintala, S. Unsupervised representation learning with deep convolutional generative adversarial networks. arXiv preprint arXiv:1511.06434 (2015).

[19] Han, C., et al. Gan-based synthetic brain mr image generation. In: Proceedings of the 2018 IEEE 15th international symposium on biomedical imaging (ISBI 2018), pp. 734–738 (IEEE, 2018).

[20] Arjovsky, M., Chintala, S. & Bottou, L. Wasserstein generative adversarial networks. In: International conference on machine learning, pp. 214–223 (PMLR, 2017).

[21] Wu, J., Huang, Z., Thoma, J., Acharya, D. & Van Gool, L. Wasserstein divergence for gans. In: Proceedings of the European conference on computer vision (ECCV), pp. 653–668 (2018).

[22] Lei Y (2019). Mri-only based synthetic ct generation using dense cycle consistent generative adversarial networks. Med. Phys..

[23] Nie D (2018). Medical image synthesis with deep convolutional adversarial networks. IEEE Trans. Biomed. Eng..

[24] Long, J., Shelhamer, E. & Darrell, T. Fully convolutional networks for semantic segmentation. In: Proceedings of the IEEE conference on computer vision and pattern recognition, pp. 3431–3440 (2015). 10.1109/TPAMI.2016.2572683 27244717

[25] Emami H, Dong M, Nejad-Davarani SP, Glide-Hurst CK (2018). Generating synthetic cts from magnetic resonance images using generative adversarial networks. Med. Phys..

[26] He, K., Zhang, X., Ren, S. & Sun, J. Deep residual learning for image recognition. In: Proceedings of the IEEE conference on computer vision and pattern recognition, pp. 770–778 (2016).

[27] Han X (2017). Mr-based synthetic ct generation using a deep convolutional neural network method. Med. Phys..

[28] Yurt M (2021). mustgan: Multi-stream generative adversarial networks for mr image synthesis. Med. Image Anal..

[29] Kim, T., Cha, M., Kim, H., Lee, J. K. & Kim, J. Learning to discover cross-domain relations with generative adversarial networks. In: International conference on machine learning, pp. 1857–1865 (PMLR, 2017).

[30] Hertzmann, A., Jacobs, C. E., Oliver, N., Curless, B. & Salesin, D. H. Image analogies. In: Proceedings of the 28th annual conference on computer graphics and interactive techniques, pp. 327–340 (2001).

[31] Gatys, L. A., Ecker, A. S. & Bethge, M. A neural algorithm of artistic style. arXiv preprint arXiv:1508.06576 (2015).

[32] Cheng, L., Vishwanathan, S. N. & Zhang, X. Consistent image analogies using semi-supervised learning. In: Proceedings of the 2008 IEEE conference on computer vision and pattern recognition, pp. 1–8 (IEEE, 2008).

[33] He W, Xie Z, Li Y, Wang X, Cai W (2019). Synthesizing depth hand images with gans and style transfer for hand pose estimation. Sensors.

[34] Cheng J (2015). Enhanced performance of brain tumor classification via tumor region augmentation and partition. PLoS ONE.

[35] Mao, X., et al. Least squares generative adversarial networks. In Proceedings of the IEEE international conference on computer vision, pp. 2794–2802 (2017).

[36] Chen, X., et al. Infogan: Interpretable representation learning by information maximizing generative adversarial nets. In: Proceedings of the 30th international conference on neural information processing systems, pp. 2180–2188 (2016).

[37] Szegedy, C., Vanhoucke, V., Ioffe, S., Shlens, J. & Wojna, Z. Rethinking the inception architecture for computer vision. In: Proceedings of the IEEE conference on computer vision and pattern recognition, pp. 2818–2826 (2016).

[38] Simonyan, K. & Zisserman, A. Very deep convolutional networks for large-scale image recognition. arXiv preprint arXiv:1409.1556 (2014).

[39] Zhu, J.-Y., Park, T., Isola, P. & Efros, A. A. Unpaired image-to-image translation using cycle-consistent adversarial networks. In: Proceedings of the IEEE international conference on computer vision, pp. 2223–2232 (2017).

[40] Ioffe, S. & Szegedy, C. Batch normalization: Accelerating deep network training by reducing internal covariate shift. In: International conference on machine learning, pp. 448–456 (PMLR, 2015).

[41] Maas, A. L., Hannun, A. Y. & Ng, A. Y. Rectifier nonlinearities improve neural network acoustic models. In: Proceedings of the icml, vol. 30, 3 (Citeseer, 2013).

[42] Hager WW (1979). Lipschitz continuity for constrained processes. SIAM J. Control. Optim..

[43] Halmos PR, Savage LJ (1949). Application of the radon-nikodym theorem to the theory of sufficient statistics. Ann. Math. Stat..

[44] Vincent OR, Folorunso O (2009). A descriptive algorithm for sobel image edge detection. Proc. Inform. Sci. IT Edu. Conf. (InSITE).

[45] Deng, G. & Cahill, L. An adaptive gaussian filter for noise reduction and edge detection. In: Proceedings of the 1993 IEEE conference record nuclear science symposium and medical imaging conference, pp. 1615–1619 (IEEE, 1993).

[46] Yin X, Goudriaan J, Lantinga EA, Vos J, Spiertz HJ (2003). A flexible sigmoid function of determinate growth. Ann. Bot..

[47] Kingma, D. P. & Ba, J. Adam: A method for stochastic optimization. arXiv preprint arXiv:1412.6980 (2014).

[48] Dumoulin, V. & Visin, F. A guide to convolution arithmetic for deep learning. arXiv preprint arXiv:1603.07285 (2016).

[49] Liu DC, Nocedal J (1989). On the limited memory bfgs method for large scale optimization. Math. Program..

